# Innovation in Suicidology: Artificial Intelligence-Based Risk Assessment

**DOI:** 10.1192/j.eurpsy.2025.1825

**Published:** 2025-08-26

**Authors:** N. M. Szeifert, X. Gonda, C. Kerepesi

**Affiliations:** 1 Department of Sports Medicine, Semmelweis University; 2 Doctoral School of Psychology, ELTE Eotvos Lorand University; 3 Department of Psychiatry and Psychotherapy; 4 NAP3.0-SE Neuropsychopharmacology Research Group, Hungarian Brain Research Program, Semmelweis University; 5 Institute for Computer Science and Control (SZTAKI), Hungarian Research Network (HUN-REN), Budapest, Hungary

## Abstract

**Introduction:**

The use of artificial intelligence (AI) in suicide risk assessment is gaining prominence as AI algorithms are capable of processing and analyzing large volumes of data quickly. Suicide risk assessments are traditionally carried out by psychiatrists and clinical psychologists following established protocols, but AI systems can provide valuable support in this area, particularly in prevention and faster detection. Based on the collected data, AI algorithms can create predictive models that identify individuals at the highest risk. These models can take into account previous mental health disorders, suicide attempts, and other social or economic factors.

**Objectives:**

The aim of our study was to test a suicide prediction model using an XGBoost machine learning tool.

**Methods:**

We included 357 individuals, out of which 146 were psychiatric patients with a history of suicide attempts in their anamnesis, 154 were psychiatric patients without a history of suicide attempts, and 57 individuals formed the sine morbo control group. Initially, 71 individuals (test dataset) were randomly selected from the total 357, and the remaining sample (training dataset) was used to train the XGBoost machine learning tool. This training process involved optimizing and selecting the best parameters. Afterward, the final model was tested on the reserved test dataset consisting of 71 individuals.

**Results:**

During the machine learning process, we were able to very accurately predict who had a history of suicide attempts and who did not, with a high performance indicated by a ROC AUC score of 0.96. This demonstrates the model’s excellent ability to distinguish between individuals with and without suicide attempts based on the data used.

**Conclusions:**

AI systems can complement traditional methods in suicide prevention, but they cannot replace human expertise. It is also important to pay attention to ethical issues, such as data protection and the reliability of these systems. AI can be a powerful tool in predicting suicide risk if properly integrated into mental health services.

**Disclosure of Interest:**

None Declared

